# Psychosocial stress at work and cardiovascular diseases: an overview of systematic reviews

**DOI:** 10.1007/s00420-015-1019-0

**Published:** 2015-02-17

**Authors:** Alba Fishta, Eva-Maria Backé

**Affiliations:** Federal Institute for Occupational Safety and Health (BAuA), Nöldnerstraße 40-42, 10317 Berlin, Germany

**Keywords:** Systematic reviews, Work-related psychosocial stress, Job strain, Cardiovascular diseases, Overview of systematic reviews, Stress model

## Abstract

**Purpose:**

Based on information reported in systematic reviews (SRevs), this study aimed to find out whether psychosocial stress at work leads to cardiovascular (CV) morbidity and mortality.

**Methods:**

A systematic search in PubMed and EMBASE (until 2014) used a string based on PICOS components. A manual search was followed. Applying the predefined criteria, two reviewers independently screened the titles, abstracts, selected full texts, and validated their quality. Discrepancies were resolved by discussion between reviewers. Studies of low quality were excluded. Contents of enrolled SRevs were extracted by one reviewer; a second reviewer evaluated their accurateness.

**Results:**

The search resulted in 462 records. Six SRevs based on 81 studies (total population: ~1,468,670) fulfilled the inclusion criteria, four of “very good” (++) and two of “good” (+) quality. Excluded records were filed, and reasons for exclusion were documented in all cases. Different stress models were used to measure the work-related stress; the “demand-control model” was most commonly used. The two enrolled meta-analysis confirmed a modest (1.32, 95 % CI 1.09–1.59; Virtanen et al. 2013) to moderate evidence (1.45, 95 % CI 1.15–1.84; Kivimäki et al. 2006), predominantly among men, for the association between psychosocial stress at work and CV outcomes. Due to lacking information, it was not possible to give evidence on the dose–response relationship.

**Conclusions:**

Same to a SRev, an overview of SRev is used to summarize literature and identify areas in which research is needed. This overview can be used to: (a) Disseminate an up-to-date information on work-related stress as a risk factors for CV morbidity and mortality to government, health care providers, workers, and other stakeholders; (b) Encourage governments to better regulate the working conditions and consider work-related psychosocial stress as a hazardous factor that leads to CV diseases or mortality; and (c) Analyze gaps in the literature and provide a summary of research needs.

## Background

### Description of the condition

WHO (2011) reports that cardiovascular diseases (CVDs), especially coronary heart diseases (CHD), are the number one cause of premature death worldwide. About 17.3 million people died in 2008, representing 30 % of all global deaths, and almost 23.6 million people are expected to die from CVD, mainly from heart disease and stroke, by 2030. Also the estimated disability-adjusted life years (DALYs) are expected to rise from a loss of 85 million DALYs in 1990 to a loss of about 150 million DALYs globally in 2020, classifying CVDs as the leading cause of productivity loss worldwide (Perk et al. 2012). CVDs are reported to have different origins. Among other reasons, epidemiological data confirmed that exposure to work-related psychosocial stress is an important and independent risk factor that predicts heart disease including elevation of blood pressure (Chandola et al. 2008; Steptoe and Kivimäki 2012).

The etiology of CVD is multifactorial involving genetic, biologic, and psychosocial factors. It is generally accepted that working conditions, gender, and age may be associated with the development of CVDs. Moreover, perceived job stress may vary between workers in certain occupations, enterprises, and also among different occupational groups (at least between blue-collar and white-collar groups) in the same workplace. Enough evidence confirms that, especially long-term and repeated stress experiences predict CV morbidity and mortality. Chandola et al. (2008) report a dose–response relationship between the frequency of stress and CV outcomes. Short-term or acute stress might also cause CV events which is, especially hazardous among individuals with advanced atherosclerosis (Steptoe and Kivimäki 2012). Short-term psychological stress induced transient myocardial ischemia (MI) at patients with CHD (Steptoe and Kivimäki 2012), whereas long-term stress at work increased the risk of recurrent CHD events and predicted CV morbidity and mortality in middle-aged men (Steptoe and Kivimäki 2012; Ohlin et al. 2004).

### Objective of the overview

The objective of this overview is to summarize and interpret the up-to-date evidence commencing from published systematic reviews (SRevs) that answer the research question “Does psychosocial stress at work lead to cardiovascular morbidity or mortality?”. Numerous primary studies but also several SRevs highlight the importance of this association. Per definition, a SRev, attempts to collate all empirical evidence that fulfills pre-specified eligibility criteria to answer a specific research question and provides a summary of the information reported in the individual studies that fulfilled the inclusion criteria (Liberati et al. 2009). An overview of systematic review (OSRev) can follow the same principle except that it does not generate data from primary studies but from SRevs. Since each review has its specific focus, which means specific questions as well as search strategies leading, e.g., to the inclusion of studies that have not been discussed in other reviews before, an overview of the existing reviews can give a broader perspective of the existing evidence. An OSRev makes sense in case several good and up-to-date SRevs that answer the same or a similar research question exist. Several SRevs were done on this topic, but there has been no OSRev conducted yet.

Furthermore, to our knowledge, this is the first OSRev that used a rigorous systematic procedure to generate information from published up-to-date and high quality SRevs. Without a proper summary of the available literature, it is difficult to draw inferences from science to practice. Therefore, not only SRevs but also OSRevs performed in a systematic way according to predefined protocols will increasingly be seen as the key source of information for policy makers and considered as the top of the hierarchy of levels of evidence.

## Methods

### Study procedure

The study procedure consisted of the following steps: (a) formulation of a clear, specific, and structured PICOS research question, (b) determination of the systematic search strategy by defining the search terms and electronic databases, (c) literature screening by applying the predefined inclusion and exclusion criteria, (d) quality evaluation of each SRev, (e) data extraction from enrolled SRevs, (f) summary of the results, (g) discussion and interpretation of study results, and h) identification of the need for further research.

### Criteria for considering reviews for inclusion

The searching process was done following a structured approach, and the study inclusion and exclusion criteria were defined a priori (Table 1; PEROSH 2011). A search strategy was formulated to systematically seek out systematic reviews and meta-analysis that answer the research question: “Does psychosocial stress at work lead to CV diseases or mortality?”. The specification of the key research question was done by defining the ‘PICOS’ components (P-population, I-intervention or exposure, C-control or comparison group, O-outcome, and S-study design). Accordingly, we included studies on workers (P) exposed to psychosocial stress at work (E) that had an outcome of CV morbidity or mortality (according to ICD-10, codes I00–I99) including the coronary heart diseases (CHD; I70), acute or subsequent myocardial infarction (MI; I21–I22) and other acute or ischemic heart diseases (I24–I25), angina pectoris (I20), heart insufficiency (I50), but excluding cerebrovascular accidents such as strokes (I60–I69), and defined arterial hypertension (I10–I11; O) (WHO 2010). In case enrolled SRevs used an older ICD version, we converted the used code for the diagnoses into the corresponding ICD-10 code. All studies dealing with CV outcomes such as sub-clinical atherosclerosis, blood pressure described as a metric variable, and other subclinical measures as well as gestational hypertension, pregnancy-related CV diseases, heart diseases with genetic origin, and neoplasms of the CV system were excluded.

**Table 1:** Study inclusion and exclusion criteria

Inclusion criteria	Exclusion criteria
Included reviews searched systematically in at least one electronic database	Wrong research question (wrong PICO question)
Research question based on PICOS/PEOS	S systematic review
P workers	Non-systematic review
E psychosocial stress at work	Published in a non-European language
O CV morbidity or mortality	Published before year 2000
S systematic review	Animal and human experimental studies
Language articles published in a European language	
Publication year articles published after year 2000	

Exposure to several work-related factors such as physical, chemical, biological, and psychological factors can lead to stress. In this study, we focused exclusively on the exposure to so-called psychosocial stressors at work including job insecurity. These stressors are extremely diverse and can be very different depending on the type of job. Studies dealing explicitly with exposure to bullying, precarious employment relationship (such as type of contract and duration of employment), shift work, or emotionally stressful work were excluded. Non-systematic reviews such as narrative reviews and publications where a full text was not available were excluded. We have restricted our search to reviews that were written in one of the European languages. Studies published before year 2000 were also excluded as they could be outdated. Studies that based their results on animal experimentation were excluded (PEROSH 2011).

### Searching methodology for identification of reviews

Following the OSH Evidence criteria for searching systematic reviews (PEROSH 2011), the systematic literature search was carried out in two relevant electronic databases (MEDLINE and EMBASE). One author (AF) ran the search for the period 01 January 2000–6 January 2014. For the search in MEDLINE (via PubMed), based on the PICOS question, we defined the search terms and prepared a “specific” search string (Schaafsma et al. 2006; Verbeek et al. 2005; Mattioli et al. 2010) which was then translated and used for the search in the EMBASE database (Table 2) .

**Table 2:** Search string for the MEDLINE via PubMed search

(((((occupation* OR worker*) OR (occupational diseases [MH] OR occupational exposure [MH] OR occupational medicine [MH] OR occupational risk [TW] OR occupational hazard [TW] OR (industry [MeSH Terms] mortality [SH]) OR occupational group* [TW] OR work-related OR occupational air pollutants [MH] OR working environment [TW])) AND (((psychosocial[All Fields] AND (“Stress”[Journal] OR “stress”[All Fields])) OR (“stress, psychological”[MeSH Terms] OR (“stress”[All Fields] AND “psychological”[All Fields]) OR “psychological stress”[All Fields] OR (“psychological”[All Fields] AND “stress”[All Fields]))) AND (“cardiovascular diseases”[MeSH Terms] OR (“cardiovascular”[All Fields] AND “diseases”[All Fields]) OR “cardiovascular diseases”[All Fields]))) AND (meta-analysis as topic [mh] OR meta-analysis[pt] OR meta-analysis [tiab] OR review[pt] OR review [tiab]) NOT (letter[pt] OR editorial[pt] OR comment [pt]) NOT ((animals [Mesh:noexp]) NOT (humans [Mesh]))) AND (“2000/01/01”[PDAT]: “2014/01/6”[PDAT]))	

### Data collection and analysis

**Fig. 1:**
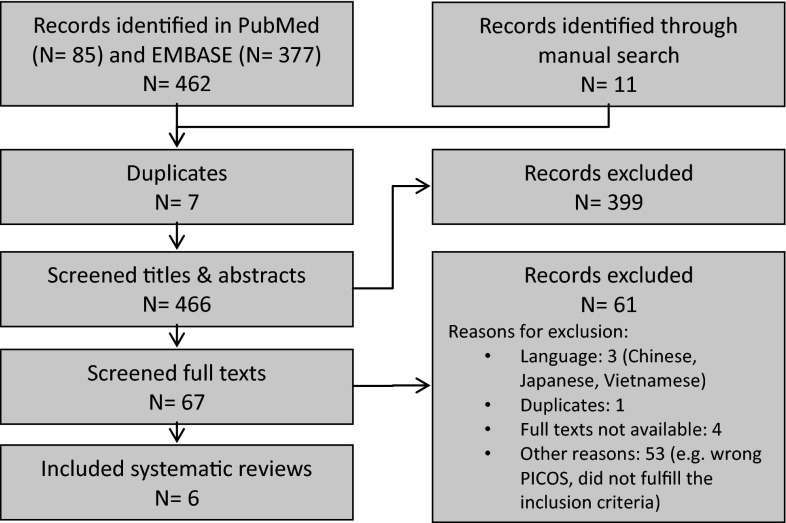
Flow diagram of the process of literature search and identification of SRevs eligible for inclusion in the OSRev

Initially, two reviewers (AF and EMB) screened the titles and abstracts of the identified literature independently from each other and eliminated irrelevant papers which did not fulfill the predefined criteria. The final study selection was based on their full texts and was done again blindly by two reviewers (AF and EMB). In both steps, discrepancies were solved by discussion between the two reviewers and reasons for exclusion were documented in all cases. The results of the selection process are summarized in a PRISMA diagram (Liberati et al. 2009; Fig. 1).

### Assessment of the methodological quality of included systematic reviews

Once the papers have been selected, a quality assessment of the methodology of each retrieved SRev was made by two reviewers independently (AF and EMB) using an expanded version of the Scottish Intercollegiate Guidelines Network (SIGN) checklist for systematic reviews and meta-analyses (SIGN 2012). Discrepancies were solved by discussion.

Reviews at the highest quality and with very low risk of bias or confounding are scored as “++”. In this case, according to OSH Evidence Methods (PEROSH 2011), the SRev or meta-analysis was scored as “well covered” or “adequately addressed” for all five SIGN questions and at least two questions were scored as “well covered”. SRevs with a good quality and low risk of bias or confounding are scored as “+”. This means that the review was scored as “adequately addressed” or “well covered” for at least three of the five SIGN questions. The systematic reviews with low quality and high risk of bias or confounding scored as “−” are excluded; they were scored as “adequately addressed” for three or less than three of the five SIGN questions. All quality assessment checklists are documented.

### Data extraction and synthesis

One reviewer (AF) systematically extracted the following data from all six included SRevs: population (sample size, gender), exposure, outcome, study design of studies included in the review, search strategies (searched databases, follow-up and searching period), results with regard to exposure and outcome/s, methodological quality assessment that was used to evaluate the quality of enrolled studies, methods used to validate the association between exposure and outcome/s (including the used stress model to measure the exposure), potential biases and the funding source. An additional reviewer (EMB) did a quality check of the data extraction.

### Methods to measure stress exposure

It remains important to use appropriate methods for measuring stress. The increasing concern that psychological conditions and social factors at work influence the worker’s well-being has led to the detection of several risk factors and development of epidemiological theoretical models. The extent to which work stress and health outcomes correlate with each other varies depending on the model used to measure exposure to work characteristics. The first attempts to measure job-related stress and CVD started in 1960s by developing numerous tools such as questionnaires and interviews (Landsbergis et al. 2000). The main theoretical models used nowadays to describe stress at the workplace are the demand-control or the job strain (JS) model (Karasek et al. 1998; Karasek 1979; Karasek and Theorell 1990) and the effort-reward imbalance (ERI) model (Siegrist et al. 1990; Siegrist 1996). Further dimensions are also being used to evaluate work-related stressors.

The *demand*-*control model* (Karasek and Theorell 1990) is based on psychosocial characteristics of work. The model is being used in relation to CVD in numerous epidemiological studies and now operates with three main dimensions: (a) psychological job demands, (b) job control or decision latitude, and (c) social support at work. According to this model, workers with jobs characterized by high psychological demands in terms of workload, low control over working conditions (decision latitude), and lack of social support at work (isostrain) are assumed to have the highest risk of poor psychological well-being and ill health. On the other side, high control and low demand are the most beneficial to health (Karasek and Theorell 1990). Due to the fact that social support at work has shown to modify the strain that might lead to stress, some studies evaluate the dimension of social support in combination with the JS model (the so-called isostrain model; Johnson et al. 1989). Most studies use two measures of social support: the supervisors support and co-workers support. A Job Content Questionnaire (JCQ) is used to measure job stressors.

The *effort*-*reward* *imbalance* (*ERI*) *model* (Siegrist et al. 1990; Siegrist 1996) is an alternative and important model that is being used in occupational health research to evaluate stress. The model is based on the premise that work-related stress happens due to lack of reciprocity at work. Siegrist et al. define threatening job conditions as a mismatch between efforts or high workload (high demand) and low control over long-term rewards consisting of money, esteem, and job security or career opportunities (Siegrist et al. 1990; Siegrist 1996). In summary, according to the ERI model, work characterized by both high efforts and low rewards in terms of salary, esteem, or job security represents a reciprocity deficit between ‘‘costs’’ and ‘‘gains’’. Working hard without receiving appreciation is an example of a stressful imbalance that increases health problems. Additionally, to effort and rewards, the ERI model includes a third component—overcommitment—which refers to a set of attitudes, behaviors, and emotions reflecting excessive striving in combination with a strong wish to be approved and esteemed.

The *Organizational Justice* (*OJ*) *dimension* defines the quality of social interaction at work and evaluates the decision-making rules and managerial behaviors within the organization. It is used to evaluate the extent to which people perceive that they are treated fairly by their supervisors and assumes that stress-related disease happens because the individual does not feel treated fairly in the organization (Elovainio et al. 2010). The dimension evaluates the extent to which employees are treated justly and whether the outcomes obtained and the processes carried out at the workplace are fair. In this case, workers seem to be affected not only by rewards as such, but also by the procedures used to determine how they will be distributed (Elovainio et al. 2002). Originally, the dimension was used to evaluate the distribution of justice and perception of equity (Elovainio et al. 2010).

Besides stress described in these models, there are also further factors, e.g., job insecurity that contributes to the perception of stress at the workplace (Table 5).

## Results

### Literature search for identification of reviews

All 473 search matches (MEDLINE: *N* = 85, EMBASE: *N* = 377, hand searching: *N* = 11) were merged and stored in the literature database Reference Manager 12. After eliminating the duplicate references, titles and abstracts of the remaining publications (*N* = 466) were screened and full texts for all articles not eliminated at this step were obtained. After a full text screening (*N* = 67), only six SRevs met the prerequisites to be included in this OSRev. The list of excluded studies can be obtained by contacting the authors. The study identification process is illustrated in Fig. 1 using the PRISMA flow diagram for reporting SRevs and meta-analyses (Liberati et al. 2009; Moher et al. 2009).

### Description of included reviews

The epidemiologic evidence is derived from six SRevs (Belkic et al. 2004; Netterstrøm and Kristensen 2005; Kivimäki et al. 2006; Eller et al. 2009; Backé et al. 2012; Virtanen et al. 2013) and includes a total of 81 studies and a population of approximately 1,468,670 individuals (Table 5). All SRevs measured the relationship between psychosocial conditions at work and CV morbidity and mortality. In two SRevs, a meta-analysis was conducted (Kivimäki et al. 2006; Virtanen et al. 2013). Generally, three different measures of exposure were considered: (a) objective evaluation of the stressors, (b) subjective or self-reported stressors, and (c) the so-called ecologic method (Walter 1991a, b). Although different models were used, all reviews considered the exposure to psychosocial stress at work and comparable cardiovascular outcomes. There are enough similarities between the studies to combine them in a reasonable way.

Due to the fact that enrolled SRevs answered the same or similar PICOS question(s) and that their period of literature searching overlapped partially, to some extent they were based on the same primary studies. However, they differed from each other in aspects such as quality, methodology used for measuring the exposure, considered outcomes, criteria used to include and exclude studies, and also in the interpretation of the results they found. The degree of overlap in studies and population is presented in Table 3 (the original data analyzed for this matrix can be obtained by contacting the authors).

**Table 3:**
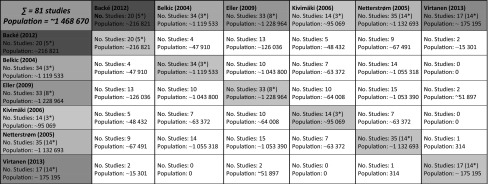
Overlap matrix of studies and considered populations

Studies marked (*) can only be found in this systematic review

All SRevs report that consistent evidence confirms a significant association between psychosocial stressors at work and CV morbidity or mortality. Due to lacking studies, this association was confirmed mainly among men and only some (not entirely consistent) evidence was found among women. The strength of the association was dependent on the methods or models that were used to measure and evaluate stress-related conditions at work as well as the target population or population subgroups that were examined. All SRevs disclosed a number of significant and nonsignificant trends toward associations and showed a large variation in the measurements of exposure and study designs. Working under high strain compared to low strain causes a significant risk of developing a CVD; a modest to moderate association was reported (risk estimates: 1.33–2.62; Backé et al. 2012). A modest association was reported for high effort versus low reward (RR 1.58, 95 % CI 0.84–2.97; Kivimäki et al. 2006) as well as for perceived job insecurity and incident CHD (RR 1.32, 95 % CI 1.09–1.59; Virtanen et al. 2013).

### Quality validation of included reviews

The methodological validation of the six SRevs included in this OSRev, according to our criteria, showed that they varied in their quality (Table 4). Four SRevs were of highest quality and limited risk of bias (++) (Belkic et al. 2004; Netterstrøm and Kristensen 2005; Backé et al. 2012; Virtanen et al. 2013), and two SRevs were of high quality and low risk of bias (+) (Eller et al. 2009; Kivimäki et al. 2006). SRevs of low quality and high risk of bias (−) were excluded.

**Table 4:** Study validation according to SIGN Quality Assessment for Systematic Reviews and Meta-Analyses

Enrolled SRev First Author, Publication year	1 Internal validity					2. Overall grading of the study quality (++/±)
	1.1 An appropriate and clearly focused question	1.2 A description of the methodology used	1.3 Sufficiently rigorous literature search to identify all relevant studies	1.4 Assessment of the study quality	1.5 Similarities between the studies selected to combine them reasonably	
(Belkic et al. 2004)	Well covered (A clear, specific and well-defined question is addressed)	Adequately addressed (description of the data synthesis is missing)	Adequately addressed (a systematic search is done in MEDLINE and with manual searching. Additional databases could be searched)	Well covered (quality evaluation of included studies is done)	Well covered (there are enough similarities between the selected studies to justify combining them; the research question is answered)	++
(Netterstrøm and Kristensen 2005)	Adequately addressed (PICO elements could be more specific)	Adequately addressed (data synthesis and study inclusion and exclusion criteria are not described)	Adequately addressed (a systematic search is done in MEDLINE and with manual searching. Additional databases could be searched)	Well covered (quality evaluation of included studies is done)	Well covered (there are enough similarities between the selected studies to justify combining them; the research question is answered)	++
(Backé et al. 2012)	Well covered (a clear, specific and well-defined question is addressed)	Adequately addressed (data extraction and description of the data synthesis is missing)	Well covered (a systematic search is done in MEDLINE, Cochrane Library, EMBASE, PSYNDEX, PsycINFO and with manual searching)	Well covered (quality evaluation of included studies is done)	Well covered (there are enough similarities between the selected studies to justify combining them; the main research question are answered)	++
(Eller et al. 2009)	Adequately addressed (PICO elements could be more specific. The used search string or search terms are not available)	Adequately addressed (description of the data synthesis is missing)	Adequately addressed (a systematic search is done in MEDLINE and with manual searching. Additional databases could be searched)	Adequately addressed (quality evaluation of included studies is done; a sensitivity analysis to exclude the studies at low quality is not done)	Adequately addressed (the study inclusion and exclusion criteria are not clearly described in order to evaluate whether the selected studies are similar to justify combining them)	+
(Kivimäki et al. 2006)	Adequately addressed (PICO elements could be more specific)	Adequately addressed (quality assessment is not described)	Adequately addressed (the systematic search is only done in MEDLINE and manual searching. Additional databases could be searched)	Poorly addressed (quality evaluation of included studies is not there)	Well covered (there are enough similarities between the selected studies to justify combining them in a meta-analysis; the research question is answered)	+
(Virtanen et al. 2013)	Well covered (A clear, specific and well-defined question is addressed)	Well covered (a detailed description of the methodology used is included)	Well covered (a systematic search is done in MEDLINE, EMBASE, Web of Science and manual searching)	Adequately addressed (quality evaluation of included studies is not mentioned)	Well covered (there are enough similarities between the selected studies to justify combining them; the research question is answered)	++

Three SRevs (Netterstrøm and Kristensen 2005; Kivimäki et al. 2006; Eller et al. 2009) had a rather broad research question; the PICO question or its elements could have been more specific and well-defined to achieve higher quality. With regard to the methodology, description of the data extraction and data synthesis was missing in four SRevs (Belkic et al. 2004; Netterstrøm and Kristensen 2005; Eller et al. 2009; Backé et al. 2012). Furthermore, a good SRev should use clear criteria to assess whether individual studies were well conducted before deciding whether to include or exclude them (SIGN 2012). One SRev (Kivimäki et al. 2006) did not report to have done the quality validation of included studies. Netterstrøm and Kristensen (2005) included in their SRev studies of low quality (29 of the 35 studies included achieved a quality score of 16 from 25 points). In another SRev (Eller et al. 2009), the studies were evaluated for their quality but a sensitivity analysis to exclude studies at low quality was not done.

Four SRevs (Belkic et al. 2004; Netterstrøm and Kristensen 2005; Kivimäki et al. 2006; Eller et al. 2009) additionally limited their literature search in the MEDLINE database to manually searching key journals and following up reference lists of included studies. Searching in further relevant electronic databases such as EMBASE, PSYNDEX, and PsycINFO could have been done to decrease the probability of missing important literature. Only in one SRev (Eller et al. 2009), the inclusion and exclusion criteria were not clearly described in order to be able to evaluate whether the selected studies are similar and therefore easily combinable. In the remaining five SRevs (Belkic et al. 2004; Netterstrøm and Kristensen 2005; Kivimäki et al. 2006; Backé et al. 2012; Virtanen et al. 2013), the key research question was fully answered and there were enough similarities between the studies selected to justify combining them.

### Main results with regard to the research question and its PICOS elements (see also Table 5)

#### Study population (P)

The association between job stress and CV outcomes was consistent among men, and some evidence (not entirely consistent) was found for women. Most studies included in each SRev involved only men; there were too few studies on women to draw conclusions (Backé et al. 2012; Eller et al. 2009). A gender and age difference was reported in individual studies (Chandola et al. 2008) but, due to a lack of available investigations where gender stratification was done, no conclusions were drawn in the enclosed reviews. For example, in their SRev, Kivimäki et al. (2006) report that a complete test of gender differences in the association between job stress and CHD was not possible because there were only two studies available that report only risk ratios for both men and women in combination. Belkic et al. (2004) report that women are more likely than men to have low levels of control over their work, and the ones working in jobs with low decision latitude are expected to have higher psychological demands. Therefore, women are several times more likely than men to hold high-strain jobs, whereas men’s high-demand jobs are generally to some extent accompanied by higher decision latitude. Furthermore, women (the same holds for men) could be stressed for other reasons such as unpaid work at home, changing hormones, home and family responsibilities, or the marital status. For example, based on individual studies, Kivimäki et al. (2006) report that low control at home predicts CHD among women but not among men and a combination of stress at home and at work predicts perceived symptoms among women. Given that CVD is by far the biggest cause of death in women (Perk et al. 2012) and that women develop CHD later in life than men (Conroy et al. 2003), using subclinical disease measures as tools to examine psychosocial hypotheses in women earlier in the pathogenesis of CHD is crucial (Low et al. 2010).

**Table 5:**
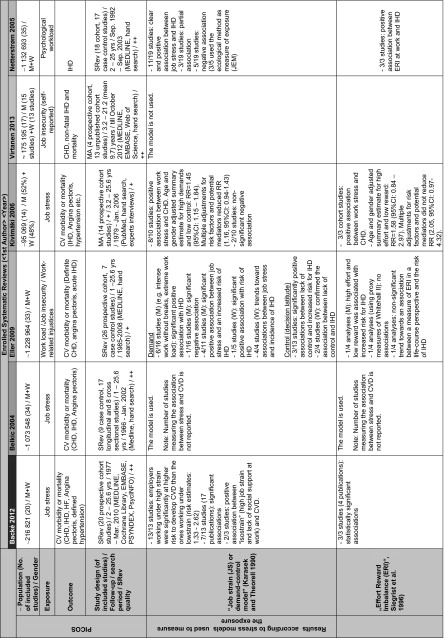

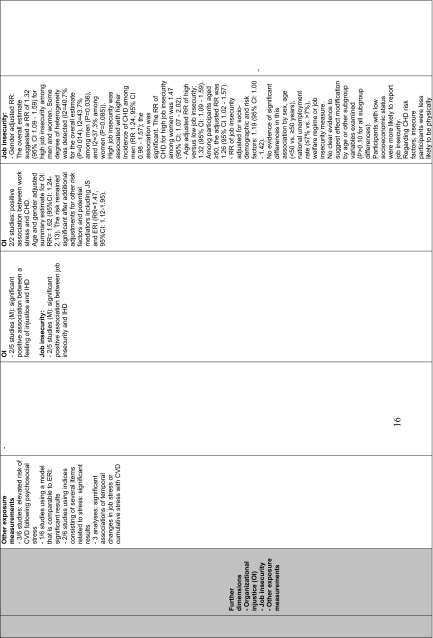

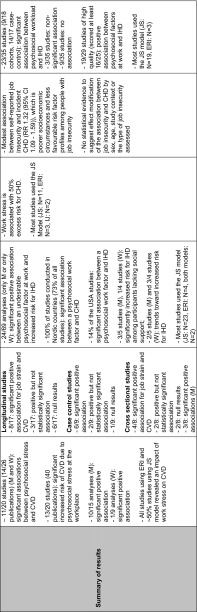
Characteristics of the original systematic reviews

*M* men, *W* women, *JS* job strain, *CHD* coronary heart disease, *CVD* cardiovascular disease, *HF* heart failure, *IHD* ischemic heart disease, *MA* meta-analysis, *MI* myocardial infarction, *IHD* ischemic heart disease, *SRev* systematic review, *Yrs* years

Significant heterogeneity in psychosocial stress was a problem of the study population enrolled in all studies included in each SRev. Backé et al. (2012) report that most of the studies enrolled in the SRev have not been specifically designed to answer the question whether there is an association between work stressors and CV outcomes.

Only three out of six enclosed reviews report on the influence of cultural variation and country differences (Eller et al. 2009; Kivimäki et al. 2006; Netterstrøm and Kristensen 2005). Most studies were conducted in the US and Europe (UK, Denmark, Germany, Sweden, Spain, Belgium, and Finland, and most of them in the Nordic countries); one study was performed in Asia (Japan). Eller et al. (2009) report that all studies conducted in the Nordic countries report a positive and significant association between job stress and CHD. All five US studies enrolled in the SRev of Netterstrøm and Kristensen (2005) report no or only partial association between psychosocial job factors and ischemic heart disease (IHD). The 11 studies showing a positive association were carried out in the UK, Sweden, the Czech Republic, and Denmark (Netterstrøm and Kristensen 2005).

#### Exposure measures (I)

To evaluate the characteristics of the workplace and to analyze the most important job stressors, studies enrolled in each SRev used the theoretical models, a modification of these models or further dimensions. Five SRevs (Backé et al. 2012; Belkic et al. 2004; Eller et al. 2009; Kivimäki et al. 2006; Netterstrøm and Kristensen 2005) report that the mostly used stress model was the job strain Model (Karasek et al. 1998; Karasek 1979; Karasek and Theorell 1990). Using the JS model, compelling evidence showed that low decision latitude is predictive for future CV morbidity and mortality. In the SRev of Belkic et al. (2004), eight investigations overall showed significant positive results with effect sizes (range for men: OR = 1.21, 95 % CI 1.08–1.35–4.0, 95 % CI 1.1–14.4; range for women: 1.3, 95 % CI 1.1–1.6–SMR = 164, 95 % CI 112–233). Limited data on *workplace* interventions aiming to increase decision-making latitude or diminish psychological demands (e.g., by reducing time pressure) showed favorable changes in mediators relevant to the CV system (e.g., blood pressure or the catecholamine and lipid profile; Belkic et al. 2004). Social support was, in consensus, reported in all SRevs as a potential confounder for job stress, and the association between stress at work and CVD could have been influenced by the social class which would act as an effect modifier (Belkic et al. 2004).

None of the enclosed SRevs report on the duration of exposure. Exposure was mostly measured at one point in time only. Generally, there was limited evidence available regarding exposure in specific occupations, and there was no evidence for specific occupational groups. Insufficient evidence was reported for a relationship between the IHD and job stress using ERI (Backé et al. 2012; Belkic et al. 2004; Kivimäki et al. 2006; Netterstrøm and Kristensen 2005), and IHD and injustice, or long working hours (Eller et al. 2009). In contrast to Eller et al. (2009), the recent review of Virtanen et al. (2013) reports a modest correlation between job insecurity and CVD (RR 1.32, 95 % CI 1.09–1.59; Table 5).

#### Outcomes (O)

Since most of the studies included in SRevs investigated CVD as a whole, it was not possible to evaluate whether job stress acts differently in relation to specific outcomes alone, e.g., myocardial infarction (MI), angina pectoris, hypertension, or stroke within the same study population. One review (Backé et al. 2012) reports significant results for six out of 14 publications investigating CHD and for five out of seven articles on CVD. One of the two publications on hypertension, one of the two publications on stroke, and one publication on angina pectoris revealed statistically significant positive associations. One SRev (Eller et al. 2009) reports that sudden death was included as an end point in some studies, but it does not always happen due to MI. Arrhythmia may very well be originated by the same conditions as ischemic heart disease on the basis of atherosclerosis; however, the time from exposure to outcome may be much shorter (Eller et al. 2009).

Work stress also has an impact on CV re-events (e.g., after MI) (Aboa-Éboulé et al. 2011) or on the prognosis of other CVD. None of the enrolled SRevs analyzed the prognostic association between work stress and CVD. A reason could be the incompleteness of data available. Backé et al. (Backé et al. 2012) argued that detailed job descriptions were lacking in most of the studies included in the SRevs.

## Discussion of quality, overall completeness, and applicability of evidence

### Interaction mechanisms

Multiple patho-physiological mechanisms which stimulate vascular inflammation and atherosclerosis are involved in the stress response (Black 2006). These mechanisms activate the autonomic nervous system and might deregulate the hypothalamic pituitary adrenal axis resulting in increased adrenaline and cortisol secretion patterns and development of the metabolic syndrome (Chandola et al. 2008; Black 2003). The CV system is consequently prone to: (a) increased heart and blood flow rate due to which the heart might retain an abnormal rhythm or problems of its muscle, (b) elevated blood pressure due to which the CV system can experience all common problems that are associated with hypertension including damaged blood vessels, accelerated atherosclerosis, and increased risk of heart disease and stroke, and (c) higher cholesterol and triglyceride levels in the bloodstream which increases the risk of plaque and thus could lead to coronary artery disease (CAD) or heart attack (Lewington et al. 2002). In summary, in certain individuals, stress leads to deterioration of the CV system “directly” through activation of neuroendocrine stress pathways and initializing atherosclerosis or “indirectly” through unhealthy lifestyle behaviors such as smoking, alcohol consumption, diet, and lack of physical activity which encourage CV risk factors such as high cholesterol level, overweight, poor dietary habits (Perk et al. 2012).

This OSRev confirmed that work-related stress is an important social determinant of CV diseases and mortality. However, there is little longitudinal evidence on the mechanisms which cause cumulative stress at work to affect an employee’s health. The way how stressful conditions are translated into changes in disease patterns is also not completely clear in the evidence (Perk et al. 2012). Such mechanisms and the psychosocial pathways that might mediate the effect of exposures to job stress should be the object of more theorization and testing in the future (Chandola et al. 2008; Eller et al. 2009).

Work life is in continuous change; studies on work life changes are lacking (Kivimäki et al. 2012). An example of such changes is organizational downsizing due to which increases in JS and ERI among those who keep working is expected (Kivimäki et al. 2012). Moreover, it is advisable to jointly consider work and out-of-work-related conditions that encourage stress. However, due to insufficient evidence, more research is needed to evaluate also single risk factors (Kivimäki et al. 2012). Potential stress factors such as the low socio-economic status, lack of social support, crisis or conflicts in family life (Eaker et al. 2007), bullying at work, depression, anxiety, hostility, type D personality (Perk et al. 2012; Backé et al. 2012), genetic predisposition, and financial strain could contribute to the development of CVD, worsening of clinical course, and their prognosis. The meta-analysis of (Kivimäki et al. 2006) showed that the association between work-related stress and CHD significantly decreased after adjustment for covariates, such as socioeconomic position, body mass index, blood pressure, cholesterol concentration, smoking, and sedentary lifestyle. Employees experiencing chronic work-related stress and who, in addition, are socially isolated or lonely have an increased risk of a first coronary heart disease event (Steptoe and Kivimäki 2012). Furthermore, consideration of other work-related factors (e.g., noise, cold, physical workload, shift work, overtime work, exposures to toxic chemicals) and the enquiry of several lifestyle factors and interactions between risk factors would allow developing new concepts concerning the multifactorial etiology and prevention of CVD (Backé et al. 2012; Kivimäki et al. 2012). Such data need to be stratified for potential effect modifiers such as age groups, gender, occupation, and occupational group. Based on only two case control studies, Belkic et al. (2004) report that shift work does not confound the association between job stress and MI. Although the importance of long working hours is emphasized in several observations considered in all SRevs, none of the SRevs adjusted for risk estimates for working long hours in a high-strain job. According to Belkic et al. (2004), physical factors such as heavy lifting, vibration, noise, and extreme heat or cold are considered potentially harmful to the CV system (especially as possible trigger mechanisms). However, seems that little evidence linking these exposures to hard CV outcomes is available.

The correlation between exposure to job strain (conflicting demands, work pace, and decision latitude) and CV morbidity or mortality could be due to several non-causal mechanisms which include confounding by negative affect, health behaviors, or social class additionally to reverse causation where individuals with underlying poor health may rate their jobs as more stressful (Frese and Zapf 1988). According to the Whitehall II study, greater work stress (self-reported) was associated with poorer health behaviors in terms of eating less fruit and vegetables or less physical activity (Chandola et al. 2008). On the other hand, the long latency period between exposure to some distant risk factors and development of CVD as well as the multi-etiological character of CVD makes the differentiation between individual causal risk factors or risk markers difficult (Kivimäki et al. 2006).

### Groups at higher risk

Attempts to prevent CVD started decades ago by suggesting two different approaches to etiology, respectively, in benefit of the population as a whole (seeking to control the determinants of CVD incidence) and the population at risk (intending the individual protection) (Rose 1985). The European Guidelines on CVD Prevention in Clinical Practice suggests that preventive efforts should be lifelong—from birth to old age and recommends stress management programs at least for individuals at high risk (Perk et al. 2012). It is, however, not easy to identify the groups at higher risk since the same environmental stressors are unlikely to induce similar stress reactions in the entire population. For these reasons, risk profiles for people exposed to work stress need to be established and validated in future studies (Kivimäki et al. 2012) followed by intensive public health and individual preventive efforts (Perk et al. 2012). Among the psychosocially stressed working population, some, but insufficient, evidence is available and obviously more primary research is necessary for risk groups such as *elderly and young employees* where consideration of gender, occupation, and occupational level is crucial. There is also little evidence on the benefit these prevention programs convey, especially for the population at high risk and in a specific *occupation* or *occupational group*. More stress management intervention studies are needed to validate the benefits of such proposed programs.

### Availability of evidence on job stress interventions

There is a clear need for primary large-scale work stress intervention studies with long follow-up periods aiming to examine the effects of new actions for lowering work stress and changing the work organization (e.g., changes related to demands, decision-making latitude, and quality of leadership). An example is the reduction of working time, which has shown favorable changes in mediators relevant to the CV system such as blood pressure or the catecholamine and lipid profile (Belkic et al. 2004). The best way to measure the success of interventions would be to measure subclinical changes rather than long-term outcomes such as CV mortality (Backé et al. 2012). Such intervention studies may improve the understanding of both causality and means of prevention (Kivimäki et al. 2012).

### Age aspect

Length of exposure would be important, especially, for the younger employees because they often perceive stressors as more uncontrollable, and in contrast to the elderly, this population group is less likely to be under the effect of other risk factors (Eller et al. 2009). Consequently, more studies on younger population groups would be advisable in order to make the negative influence of psychosocial working conditions on health more evident. Aging workers were part of the target population in all SRevs, which could increase the risk of “healthy worker effect”. In his meta-analysis, Kivimäki et al. (2006) estimated the age and gender-adjusted relative ratio of CHD for high versus low job strain to 1.43 (1.15–1.84). In another study (Kivimäki et al. 2012), when participants of all ages were included, the HR was 1.35, but when only those aged 19–55 at baseline were included, it rose to 1.82.

### Gender differences

The nature of job exposures and patterns of CV manifestation and age-related prevalence is also highly gender specific (Belkic et al. 2004). Gender is certainly a critical further effect modifier for which stratified analysis is essential. Most reported significant associations between psychosocial stress at work and CV outcomes came from analyses considering only men (Backé et al. 2012; Eller et al. 2009). Backé et al. (2012) concur that a generalization of study results from men to women would not be applicable because there are gender differences for the influence of job stress on CVD. Rather than merely adjusting for gender, studies measuring the association between job stress and CV outcomes could test hypotheses separately for men and women, either through stratified analyses or by testing interactions with gender. Stress perception may also be different for women than for men. Mediators such as marital strain, family responsibilities, and strain due to multiple roles or lack of reciprocal supportive relationship may be particularly significant for women only, while work-related stress may be less important.

### Impact of the culture and geographical diversity

Among other risk factors, culture and country of origin of the target population might also have an effect on the association between stress at work and CV outcomes. Three SRevs enrolled in this overview (Eller et al. 2009; Kivimäki et al. 2006; Netterstrøm and Kristensen 2005) report that the studies they considered were mainly conducted in the United States and Europe. The meta-analysis (Kivimäki et al. 2012) noted differences in the effect of job stress on CHD between studies from Nordic countries, continental Europe, and the UK. The information was, however, insufficient to conclude whether a cultural variability existed. Due to such variability, a degree of misclassification may happen in countries in which health care is not for free (for instance in the US). In such countries, individuals with lower incomes might not have enough financial resources to cover all necessary expenses of their treatment or hospitalization; in contrast, the ones with high incomes may be over-treated and over-examined leading to the overrepresentation or underrepresentation of the individuals with higher or lower socioeconomic status, respectively (Eller et al. 2009).

Furthermore, more than 80 % of all CV mortality occurs in the developing countries (Perk et al. 2012). The available literature is based only on developed countries. There is also lack of country-based research also differentiating developing from developed countries.

### Job stress and validity of instruments that are used to measure stress

Due to many existing and continuously improving theoretical ways to define and evaluate load and strain, measuring stress at work was not easy (Nubling et al. 2006). In this OSRev, JS was mainly measured with the help of theoretical models were the job strain model (Karasek et al. 1998; Karasek 1979; Karasek and Theorell 1990) was the mostly used model. However, the quality and consequently, the reliability on stress models is often being criticized. While external or “objective” reporting can be influenced by the subjectivity of the expert, self or “subjective” reporting is also known to be prone to bias due to over or underreporting on environmental conditions (Theorell and Hasselhorn 2005). However, it still remains questionable whether workers in identical or similar working environments respond highly similarly to the Job Content Questionnaire (JCQ) used for the job strain model (Karasek et al. 1998; Karasek 1979; Karasek and Theorell 1990). For example, the Whitehall II study found a significant relationship between low decision latitude and risk of MI when using expert reporting of the work exposure. In this study, external measures of job characteristics were associated more strongly with higher rates of sickness absence compared with self-assessed reporting for low frequency and fast work pace and lower conflicting demands (Rehkopf et al. 2010). Also Persson et al. (2012) report large variations on how workers from the same industry with similar working conditions and conducting similar work responded to the JCQ.

Although there are stress-theoretical models, a standardization in the assessment of stressors at work does not exist. A variety of measurement instruments is being used to assess work stressors among studies included in each SRev. Furthermore, many studies modified the standard questionnaires or stress scales. Kivimäki et al. (2012) report that while the measure of job strain in all of the studies using the JS model is generated by cross-tabulating the dichotomized or trichotomized scales of job demands and job control, the items included in these scales often vary between the studies. Furthermore, at present, there is no agreement whether the two dimensions of high demands or low control observed separately have stronger effects on CV health than the concept of ‘job strain’ that is based on both scales, demand, and control (Eller et al. 2009; Belkic et al. 2004). Regarding the ERI model, the item content of the scales varied between the studies, as well (Kivimäki et al. 2006). In such circumstances, the usage of models could in fact be a risk of bias itself. If you want to answer questions using a stress model, you might overlook and consequently underestimate some risks. Using the same instrument to measure the correlation between exposure and outcome is therefore advisable.

On the other hand, work life is in continuous change and therefore linked to new types of stressors that need to be considered in the theoretical stress models which are used as instruments to evaluate stress. In such a situation, updating the existing theoretical models and inclusion of further dimensions are advisable. More studies with sophisticated assessment of the development of job stress over time and its impact over health are necessary (Backé et al. 2012). The ERI model, organizational injustice, and job insecurity are examples of new theories that have to be evaluated in future studies before determining the effect of the included dimensions (Eller et al. 2009).

### Dose–response relationship between work-related stress and CVD

This OSRev confirmed that although enrolled reviews due to lacking information (in primary studies exposure was measured only in one point in time) could not measure a dose–response relationship, they agree that a correlation between work-related stress and CV morbidity and mortality exists. Belkic et al. (2004) found that employees working under same or similar conditions and are exposed to high levels of job strain (high or intermediate demands and low control) are at higher risk than the ones exposed to intermediate job strain levels. Consistent with a prospective cohort study (Kivimäki et al. 2002), Belkic et al. (2004) report indirect evidence of a temporal dose–response relationship based on a stratified analysis with workers whose occupational group did not change for over 5 years. This group of workers revealed a higher hazard ratio (HR 2.9, 95 % CI 1.25–6.71) compared to the whole cohort. Furthermore, several studies enrolled in the SRev of Belkic et al. (2004) found a dose–response effect for decision latitude alone and risk of incident CVD, but no dose–response relationship was found for CV mortality.

In general, there were few longitudinal studies examining the effect of cumulative work stress on other intermediate mechanisms despite the evidence that chronic stress predicts CV morbidity and mortality (Chandola et al. 2008; Kivimäki et al. 2006). Three SRevs (Backé et al. 2012; Eller et al. 2009; Kivimäki et al. 2006) report that most of the studies that included assessed exposure to job stress only at one point in time (only at baseline). Exposure measures at different points in time were not reported from any other included SRev. Individual studies confirmed that measuring exposure at different points in time would change the results. For example, within the Whitehall study, temporally increased exposure in men (using ERI score) was statistically significantly related to the development of angina pectoris (Chandola et al. 2005). Measuring exposure only at baseline may be sufficient when workers experience the same level of exposure to job stress during the follow-up period, but in case a negative change in exposure happened during the follow-up (e.g., due to job change), a re-evaluation of exposure would be important. After a temporary increase in work stress, the atherosclerotic condition could progress with a different (higher) rate than otherwise. A significant change in exposure occurs when individuals retire; therefore, follow-up beyond the retirement age is not advisable (Eller et al. 2009). Examining cumulative exposures and showing dose–response relationships would contribute to a causal understanding of this association.

### Diversity in professions and occupational groups

High perceived stress is seen in (a) specific *professions* such as nurses or teachers; (b) specific groups of workers in precarious employment situations such as subcontractors or temporary or leased workers, but also among the (c) high-skilled, motivated, and dedicated ones. Furthermore, there are more and more high-qualified employees struggling with high demands. Several investigations such as the large Whitehall study (Chandola et al. 2008) report that stress varied among workers in different *occupational levels* such as office-based workers (so-called “white-collar workers”) and manual workers (“blue-collar workers”). It is seen that also higher qualified workers such as managers with a secure and well-paid job work under bad conditions, e.g., under high demands, are consequently prone to all possible stress-related outcomes. None of the enrolled SRevs report on the CV morbidity or mortality due to perceived stressors among different occupational groups of the same occupation or industry. We noticed an obvious deficit in studies evaluating psychosocial conditions at specific occupations and occupational groups and CV outcomes.

### Potential biases

#### Potential biases in the overview process

Overviews of reviews are only as good as the SRevs and primary studies on which they are based; gaps or lack of consistency in this evidence will weaken the overview of reviews. Although this OSRev was performed in a systematic way by strictly following the predefined criteria and was based on a rigorous protocol developed at the outset, we realized that due to its complexity, conducting an OSRev is not easy. Using the data of existing SRevs and drawing conclusions based on their results is a complex procedure. Each enrolled SRev would generally bring an amount of bias with it, especially because they are also based on other (primary) studies, which are a source of bias themselves. As example, authors of enrolled SRevs partially used different standards and also different instruments to validate the quality of the enrolled primary studies. Subsequently, in some cases, the same studies appeared to have different quality. Although in this OSRev we applied an up-to-date methodology and even introduced new actions, e.g., the Plot of Study Overlap Matrix, we realized that the risk of combining “apples and oranges” exists.

The methodology of conducting such overviews of systematic reviews needs improvement. We were unable to recommend a specific instrument for reaching judgments, e.g., about the possible influence of the amount of study overlap over the study conclusions. A checklist to evaluate the quality of such OSRev does not exist yet either.

#### Potential biases in the search process

The systematic literature search was run in two relevant electronic databases (PubMed and EMBASE) and was followed by a manual search. Further databases were not searched which means that some relevant publications may have been missed. However, given the fact that we searched manually in addition to the two biggest and most relevant databases, we assume that we could not have missed any relevant SRevs. Furthermore, we only considered studies published in a European language which means that, although non-English studies usually publish at least the title or also an abstract in English, a small probability of having missed such studies exists. During the literature screening, we did not find such publications; therefore, we believe that the language limitation did not bias our searching results.

For the literature search, we decided to use the more “specific” search string for occupational health studies (Mattioli et al. 2010) which is capable of retrieving more than 40 % of the relevant publications (specificity of 98 %, sensitivity of 47 %). Using the “specific” string, a high proportion of articles that do not fulfill our predefined criteria are excluded, a limited number of articles but precisely fitting to our PICOS question are found and the smallest number of false positive articles is yielded.

#### Study diversity

Although studies enrolled in each SRev partially overlapped, different interpretation of the results with respect to slightly different key research question(s) is possible.

Investigations considered in all enrolled SRevs varied in reporting on exposure, outcomes, statistical models used and considered confounders such as biological and behavioral risk factors. Eller et al. (2009) report that, in some cases, studies were hard to compare, especially with regard to the different cultures, which might be a reason of unequal stress perception, and in reference to gender and age.

A meta-analysis of individual participant data (IPD) from 197,473 European men and women based on 13 cohort studies (Kivimäki et al. 2012) was not included in this OSRev, since it was not based on an SRev. The study found a significant increase in incident CHD due to job strain based on one baseline assessment. Using non-randomized observational data, it could not make any conclusions on the causality of the findings and could not exclude residual confounding as an alternative explanation for the findings.

#### Publication bias

Due to the publication bias, published studies may not truly represent all accurate studies carried out, which may alter the results of meta-analyses and SRevs of big numbers of studies on which evidence-based medicine increasingly relies. The problem may be, especially important when the study is sponsored by entities that are interested in positive results. The collaborative meta-analysis of individual participant data from 13 cohort studies on job strain and CHD incidence found a small heterogeneity in study-specific estimates (overall HR: 1.23; 95 % CI 1.10–1.37) for job strain vs. no job strain (Kivimäki et al. 2012). Furthermore, a considerable difference in the association between job strain and CHD was noticed between published and unpublished studies (respectively, HR: 1.43; 95 % CI 1.15–1.77 and HR: 1.16; 95 % CI 1.02–1.32).

In this OSRev, it was hard to avoid the publication bias because each SRev brought some risk of publication bias with it; however, all included SRevs had no conflict of interest. The sponsors of each SRev had no role in the study design, data collection, data analysis, data interpretation, or writing of the report.

#### Information bias, underestimation or overestimation of the effect

Backé et al. (2012) reported that some of the studies included in the SRev had a long follow-up duration. In such a situation, there is a risk of information bias unless job stress remains stable and employees do not change their job or experience periods of unemployment. Job change due to stress would underestimate the effect, especially in individuals at risk. In the Whitehall study, the effect of ERI on CV health indicated higher risk estimates after an average follow-up time of 5.3 years (Bosma et al. 1998) than after a follow-up time of 11 years (Kuper et al. 2002). However, the outcome of the two analyses differs Bosma et al. (1998) consider CV morbidity and mortality and Kuper et al. (2002) only CV morbidity. The possible conclusion of an underestimation of true effect estimates in long-term studies needs further investigations.

Many studies included in each SRev might be too small to detect possible associations. A recent SRev (Pejtersen et al. 2014) that aimed to update the findings of Eller et al. (2009) by applying a stricter methodology (only papers with a high statistical power were considered) suggests that measuring an association based on studies with high statistical power is important when evaluating published studies.

## Author’s conclusions

### Key messages and needs for further research

Work-related stress is an important determinant of CV disease and mortality. Thus, cardiovascular diseases caused by work stress as one risk factor beside others can be ranked as work related according to the definition of the International Labour Organisation “a disease with multiple causal agents, where factors in the work environment play a role, together with other risk factors” (European Commission 2013). When work-related stress is considered as one of several risk factors for cardiovascular diseases, there is a need to address this issue within prevention strategies offered by occupational physicians as well as general practitioners.

Further research is necessary to: (a) evaluate the mechanisms and psychosocial pathways that might mediate the effect of job stress on employee’s health, (b) evaluate the in and out-of-work factors that encourage stress separately and in combination, (c) focus on groups at higher risk and validate the newly established risk profiles of the ones exposed to psychosocial stress at work, (d) measure the exposure in different points in time, (e) consider additional and up-to-date potential stressors in the changing working environment without limiting evaluation of stress to existing stress models, (f) evaluate whether a cultural variability exists and consider the developed and developing countries separately, (g) consider the target population grouped, e.g., with regard to gender, age, occupation and occupational level, (h) improve the ways for analyzing job stressors, and (i) aim to measure the dose–response relationship between the exposure and outcome.

### Implications for policy and practice

In a nutshell, this overview can be used to: (a) disseminate an up-to-date information on work-related stress as a risk factor for CV morbidity and mortality to government, health care providers, workers, and other stakeholders; and (b) encourage governments to better regulate the working conditions and consider work-related psychosocial stress as a hazardous factor that leads to CV diseases or mortality, which would indirectly lead to improved workers` health quality.
